# EVALUATION OF COGNITION IN PRIMARY CARE (CPC): A PILOT STUDY TO INCREASE EARLY DETECTION AND MANAGEMENT OF DEMENTIA

**DOI:** 10.1093/geroni/igac059.1617

**Published:** 2022-12-20

**Authors:** Annette Fitzpatrick, Barak Gaster, Jaqueline Raetz, Basia Belza, Monica Zigman Suchsland, Karen Tracy, Benjamin Olivari, Lisa McGuire

**Affiliations:** University of Washington, Seattle, Washington, United States; University of Washington, Seattle, Washington, United States; University of Washington, Seattle, Washington, United States; University of Washington, Seattle, Washington, United States; University of Washington, Seattle, Washington, United States; GSA, Washington, District of Columbia, United States; CDC, Atlanta, Georgia, United States; CDC, Atlanta, Georgia, United States

## Abstract

Despite evidence that early detection of mild cognitive impairment and dementia leads to improved patient care, these conditions remain under-diagnosed in primary care. To address this gap at University of Washington Medicine, we have developed and pilot-tested a program to increase cognitive evaluations and improve dementia care by primary care providers (PCPs). The Cognition in Primary Care (CPC) program was designed utilizing stakeholder-selected components of the GSA KAER (Kickstart-Assess-Evaluate and Refer) Model and Toolkit (2020 Edition), developed by the Gerontological Society of America (GSA). In this presentation we will describe evaluation of the CPC Program including (a) education training; (b) use of assessment tools integrated within the electronic health record (e.g. potentially harmful medications, depressive symptoms, alcohol use, sleep apnea, etc.); (c) practice recommendations such as dedicated evaluation appointments, diagnoses and referrals, and (d) PCP use of community resources for post-diagnosis support. A total of 66 PCPs participated in the series of 3 CME educational seminars. Over 93% of participants reported the content to be highly relevant and expected to incorporate it into their practice; commitments to use specific tools were made. Analysis is underway to determine impact of the program by comparing the above metrics before and after the training as well as between trained and untrained PCPs. Results from this evaluation will inform modifications to increase utilization of the CPC Model within UW Medicine, will be used to package the program to share with other primary care systems, and will guide future enhancements to the GSA KAER Toolkit.

